# Magnetic resonance imaging (MRI) radiomics in paediatric neuro-oncology: A systematic review of clinical applications, feature interpretation, and biological insights in the characterisation and management of childhood brain tumours

**DOI:** 10.1177/20552076251336285

**Published:** 2025-04-22

**Authors:** Teesta Mukherjee, Omid Pournik, Theodoros N Arvanitis

**Affiliations:** 1 Department of Electronic, Electrical and Systems Engineering, School of Engineering, College of Engineering and Physical Sciences, 1724 University of Birmingham, Edgbaston, Birmingham, UK

**Keywords:** Radiomics, childhood brain tumour, magnetic resonance imaging, MRI, feature extraction, systematic review

## Abstract

**Background:**

Childhood brain tumours, even though rare, present with significant diagnostic and treatment challenges. Radiomics involves feature extraction that is data-driven from standard imaging modalities, such as magnetic resonance imaging (MRI). In paediatric brain tumour imaging, MRI is often preferred because it is non-invasive and avoids exposure to radiation, making it safer for children. Radiomic features reveal additional information about tumour morphology and heterogeneity. By integrating biological meaning into imaging data, this approach enhances our understanding of tumour biology, thus supporting improved classification, treatment planning and management.

**Purpose:**

This review focuses on MRI-based radiomics for the diagnosis and prognosis of childhood brain tumours. It assesses various approaches used in image pre-processing, tumour segmentation, feature extraction, and predictive model development to understand their accuracy and outcome, while it aims to understand the biological meaning and interpretation of radiomic features.

**Methods:**

A systematic review was conducted, including 559 MRI-based radiomics studies in PubMed and Engineering Village Compendex databases, following preferred reporting items for systematic reviews and meta-analyses guidelines (PROSPERO registration: CRD42024503524). Data extraction focused on age, sample size, tumour type, pre-processing techniques, segmentation methods, feature extraction, and performance metrics.

**Results:**

Nineteen studies were included, primarily addressing ependymoma (EP) and medulloblastoma (MB). Common pre-processing methods included intensity normalisation (*n* = 11) and bias correction (*n* = 4). GLCM (*n* = 12) and GLRLM (*n* = 7) were frequently used features, with LASSO (*n* = 8) and PCA (*n* = 2) as leading selection methods. SVM was the most used classification algorithm (*n* = 9), with an AUC range of 0.858–0.977. We have included the biological meaning and clinical significance of radiomic features to further understand why these insights are important.

**Conclusion:**

While challenges such as limited datasets and varied imaging protocols exist, we recommend identifying and integrating the most informative radiomic features to enhance diagnostic and prognostic accuracy in childhood brain tumours, ultimately improving patient outcomes.

## Introduction

Brain tumours are abnormal growth of cells in the human brain, affecting its regular functioning. In children, they are the most prevalent type of solid tumour, often leading to suboptimal health outcomes.^1^ Treatment and management of such tumours depend on their size, location, type, growth status, and behaviour.^2^ In children specifically, diagnosis becomes more difficult due to the rarity of the disease, the complexity of the symptoms, ongoing brain development, the need for specialised imaging, and the precision of accurately locating the tumour.^3^ Radiomics is an emerging field which enhances conventional image analysis by extracting detailed quantitative data from various radiological images. It provides insights into tumour heterogeneity and its microenvironment. It can be applied to diverse imaging modalities such as computed tomography (CT) and magnetic resonance imaging (MRI), among others.^4^ MRI is often preferred for paediatric brain tumour imaging because it is non-invasive, provides highly detailed images of soft tissues, allows multi-planar imaging capabilities, and requires no exposure to radiation, while it also prioritises patient safety.^5,6^

Integrating radiomic analysis into clinical routine practice has the potential to improve tumour subtyping, predicting treatment responses, and monitoring disease progression.^7^ However, as this area of research has shifted towards more machine learning (ML) driven feature sets, the biological meaning and interpretation of radiomic features have often been lost. This could be one of the factors limiting clinical translation. Recent efforts are being made to reconnect radiomics with biological insights into tumour characteristics to enhance its clinical relevance.^8^ Thus, the complexities of childhood brain tumours, combined with radiomics’ advanced imaging approach, present an opportunity to improve diagnosis, refine prognosis, and optimise treatment options.^9^

While recent reviews in this area of research provide valuable overviews of radiomics and artificial intelligence in paediatric neuro-oncology, our systematic review distinguishes itself in many ways. First, it offers an in-depth analysis of the radiomics pipeline, including image pre-processing, tumour segmentation, feature extraction and selection, and predictive model development. Second, it emphasises the importance of understanding the biological meaning of the extracted radiomic features and their clinical relevance. We also aim to identify any gaps in the existing literature to propose recommendations for future research and clinical implementation. Finally, our review also considers emerging challenges such as hardware implementation offering a more comprehensive and clinically relevant perspective.

## Methods

This study has been carried out according to the following stages: (i) search strategy; (ii) study protocol and selection criteria; (iii) data extraction; (iv) study quality assessment; and (v) data synthesis.

### Search strategy

The literature search was conducted in two academic databases, PubMed and Engineering Village Compendex, known for their comprehensive coverage in this field of research. Search strings used were: for PubMed, (“radiomics” OR “radiomic”) AND (“brain tumour” OR “brain tumor” OR “brain neoplasm” OR “brain neoplasms”) AND (“MRI” OR “magnetic resonance imaging”), with a filter to remove systematic reviews; and for Engineering Village Compendex ((“radiomics” OR “radiomic”) AND (“brain tumour” OR “brain tumor” OR “brain neoplasm” OR “brain neoplasms”) AND (“MRI” OR “magnetic resonance imaging”)) WN ALL. Results from Inspec and GEOBASE were excluded. Keywords such as ‘childhood’ or ‘paediatric’ were not used; instead, studies were manually reviewed at a later stage to remove those that did not primarily focus on children, while retaining information about studies that included >50% paediatric data. Only studies involving human subjects and published in English language were considered. The search process was conducted according to the guidelines mentioned in the 2020 preferred reporting items for systematic reviews and meta-analyses (PRISMA) protocol.^10^

### Study protocol and selection

**Figure 1.**
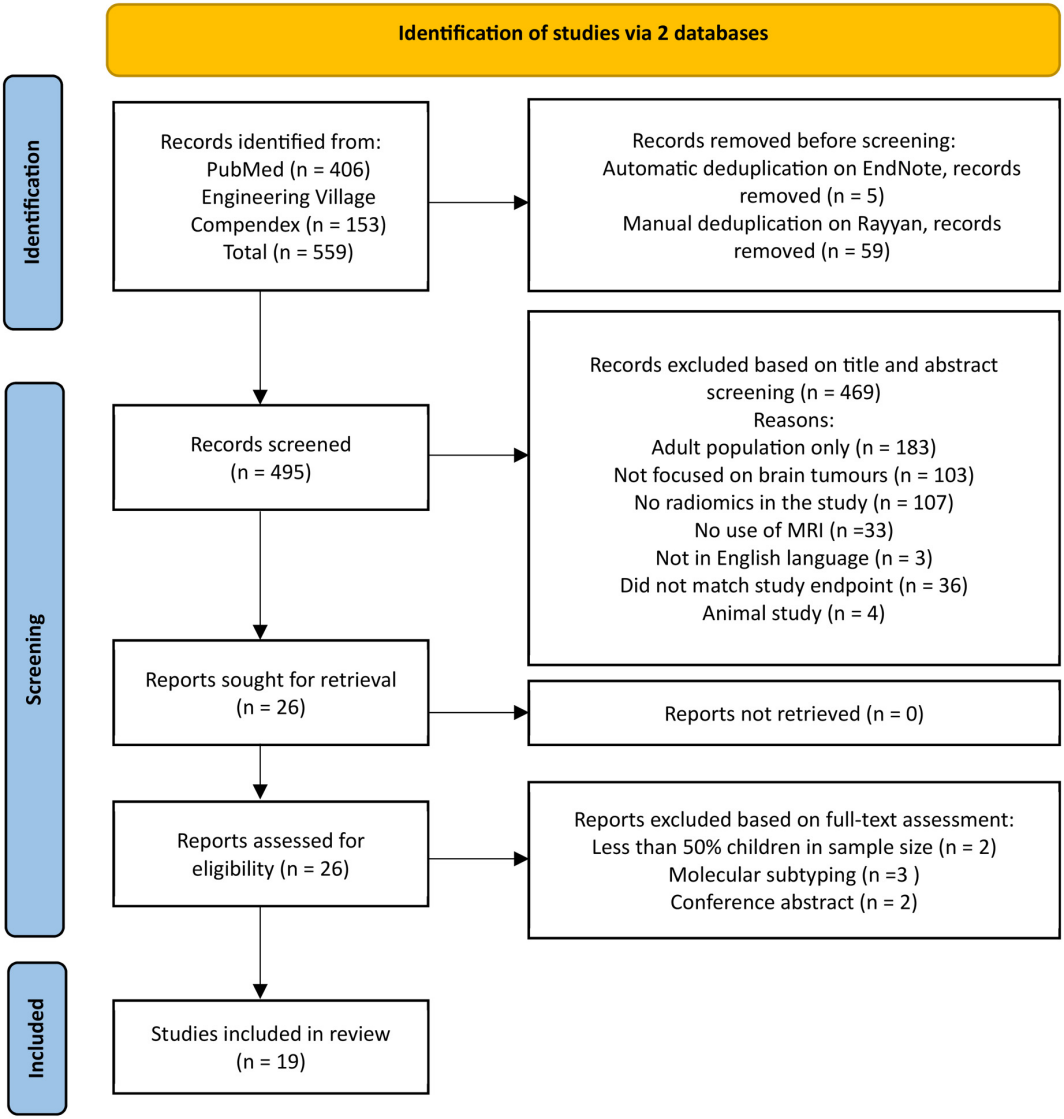
Preferred reporting items for systematic reviews and meta-analyses (PRISMA) flow diagram for study selection.

This study was registered in the Prospective Register of Systematic Reviews (PROSPERO) with protocol number CRD42024503524. The PRISMA flowchart (Figure 1) illustrates the selection process, from initial study identification to final inclusion.

The literature search was conducted up until the end of January 2025, without any initial date restrictions, to identify all relevant papers in this area of research. The inclusion criteria comprised: (i) studies focusing on radiomics, (ii) studies assessing primary brain tumours only, (iii) studies involving paediatric populations (age <18 years) or studies with >50% children cases, and (iv) studies primarily using MRI.

Exclusion criteria encompassed studies on adults, conference abstracts, and studies utilising any other imaging modality. Additionally, radiogenomics, molecular subtyping, animal-based investigations, standardisation of radiomic processes, and non-primary brain tumour studies were also excluded.

The selection process aimed at identifying studies focusing on diagnosis and prognosis in paediatric primary brain tumours. To identify studies relevant to this research question, two screening phases were undertaken: title/abstract screening followed by full-text screening. Any inconsistencies encountered during the selection process were resolved by discussion among all reviewers. When consensus could not be reached, the most senior reviewer T.N. Arvanitis (TNA) was consulted to make the final decision on whether the article met the eligibility criteria for inclusion or not.

### Data extraction

Two reviewers, T. Mukherjee (TM) and O. Pournik (OP) with expertise in radiomics and systematic review methodologies, independently conducted data extraction using a pre-determined proforma. Supervision of the study selection was provided by the senior author (TNA). Variables used for extraction of data comprised the following information: age, sample size, type of brain tumour, data collection methods, MRI sequence, image processing techniques, tumour segmentation approaches, feature extraction process, feature reduction and selection methods, predictive model development procedure, performance of the best model, and any limitations/challenges identified during the study.

### Quality assessment

Transparent reporting of a multivariable prediction model for individual prognosis or diagnosis (TRIPOD)^11^ assessment was conducted to evaluate the quality and completeness of reporting in the included studies and have been listed in Table 1. It summarises each study's TRIPOD adherence score, and comments on study objectives, internal validation, and methodologies. It also highlights areas for improvement, including insufficient calibration details and the absence of external validation in some cases. This assessment provides additional value and insights into the methodological rigour of the studies included in this systematic review.

**Table 1.** Transparent reporting of a multivariable prediction model for individual prognosis or diagnosis (TRIPOD) score.

Study reference	TRIPOD score	Comments
Dong et al.^12^	85%	Objectives and methodologies are clear; however, calibration seems incomplete, and external validation has not been performed.
Dong et al.^13^	88%	Multi-regional approach is detailed, internal validation was strong, but no external validation.
Fan et al.^14^	90%	Comprehensive methodology, both internal and external validation performed, and performance metrics have been well-reported.
Fetit et al.^15^	80%	Reporting is adequate, with limited details on model calibration.
Fetit et al.^16^	83%	Multicentre study with clear methodology; however, calibration needs more clarity.
Fetit et al.^17^	85%	Strong reporting on radiomic features; however, model calibration can be improved.
Grist et al.^18^	87%	Comprehensive multi-site study with good internal validation; however, reporting on calibration can be improved.
Li et al.^19^	82%	Clear methods and feature extraction; however, limited external validation.
Novak et al.^20^	84%	Detailed diffusion-weighted imaging approach, internal validation performed, but no external validation.
Wang et al.^21^	86%	Comprehensive methodology and calibration details can be improved.
Zhang et al.^22^	88%	Well-reported methodology and performance metrics.
Zhang et al.^23^	85%	Effective use of radiomic phenotypes; however, calibration details are underreported.
Zhang et al.^24^	87%	Comprehensive multinational study with internal validation; however, external validation is not addressed.
Zheng et al.^25^	84%	Strong focus on cerebrospinal fluid dissemination prediction but limited external validation.
Zhou et al.^26^	83%	Automated machine learning approach is clearly reported; however, calibration details can be improved.
Liu et al.^27^	86%	Well-reported survival prediction, but aspects on calibration missing.
Tam et al.^28^	88%	International study with detailed methodology; however, reporting of metrics can be improved.
Wagner et al.^29^	85%	Good reporting of radiomic features for progression-free survival; however, calibration details need improvement.
Zheng et al.^30^	90%	Comprehensive study integrating clinical and imaging features, strong reporting overall.

### Data synthesis

The selected studies showed diversity in their sample sizes, radiomic methodologies, and techniques used for segmentation and feature extraction, which did not allow for conducting a meta-analysis. Instead, this review offers a comprehensive narrative alongside a semi-quantitative summary, highlighting the application of radiomic analysis in the diagnosis and prognosis of children's brain tumours.

## Results

Our assessment of the included studies focused on various aspects related to MRI-based radiomics in the diagnosis and prognosis of paediatric brain tumours. We identified techniques used in image pre-processing, tumour segmentation, feature extraction, feature reduction and selection, and predictive model development. Accuracies achieved by the best-performing model and the biological significance of radiomic features were also evaluated. Additionally, we identified the limitations highlighted in the existing literature. These aspects have been discussed in the following sections.

### Search results

The literature search identified 559 studies from two databases: 406 from PubMed and 153 from Engineering Village. Following automatic de-duplication in EndNote,^31^ five studies were removed, and an additional 59 duplicates were removed manually. In total, 495 articles remained for title and abstract screening. Rayyan,^32^ an online tool for systematic reviews, was used to manage and screen these studies. Following this screening, 469 articles were excluded based on the pre-defined inclusion and exclusion criteria. Of the remaining 26 studies assessed in full-text, seven studies were excluded for not meeting the inclusion criteria. Thus, 19 articles were included in this systematic review.

### Study characteristics

**Figure 2.**
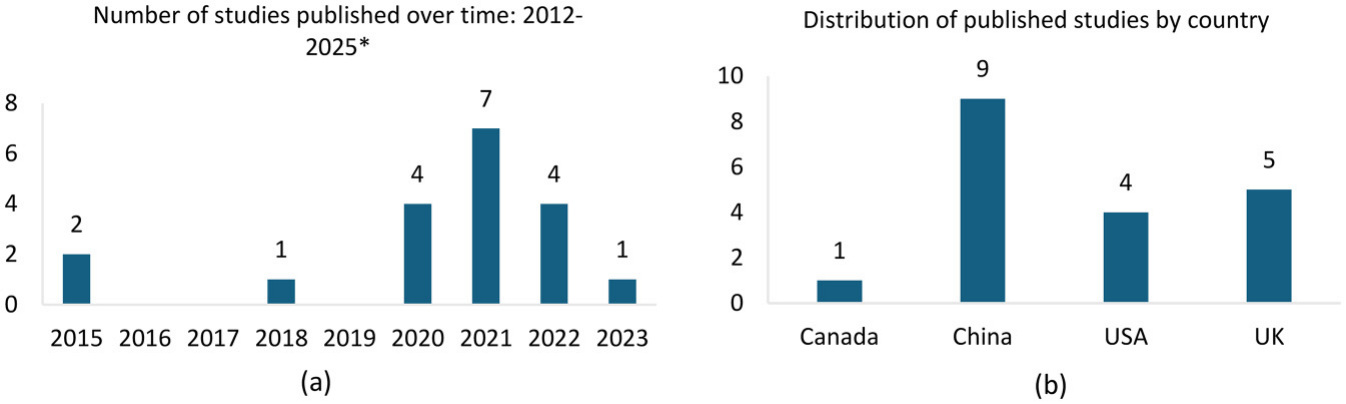
Demographics of included studies: (a) distribution of published studies over the years; (b) distribution of study geographies.

All studies included in this review were full-text articles (100%) and were retrospective (100%) in their design. The review was conducted without any time restrictions, to include all relevant studies in this field until January 2025. The distribution of publications over time and their geographical origins are shown in Figure 2(a) and (b), respectively.

A total of 10 studies (52.6%) were single centre^12–14,17,19,21,24,25,29,30^ and nine studies (47.4%) were conducted across multiple centres.^15,16,18,20,22,23,26–28^ Of the total studies analysed, 15 studies (78.9%) were diagnosis-focused,^12–26^ while four studies (21.1%) were prognosis-focused.^27–30^

The studies in this review have been categorised as either diagnosis-focused or prognosis-focused. Table 2(a) and (b) covers diagnosis: Table 2(a) includes age, sample size, tumour type, MR imaging sequence, and study limitations; while Table 2(b) contains information about image pre-processing, segmentation, feature extraction, feature selection/reduction, and model performance. Table 3(a) and (b) provides the same for prognosis.

**Table 2.** Radiomics approach for diagnosis of paediatric brain tumours.

(a)						
Study ref.	Focus	Age range (years)	Sample size	Type of brain tumour	MR imaging sequence	Limitations
Dong et al.^12^	Differentiation between EP and MB	0–15	51	EP = 24, MB = 27	T1C, DWI (ADC)	Small sample size, limited tumour region and MR sequences, lack of separate validation set
Dong et al.^13^	Classify paediatric PFTs	Median: primary cohort = 6, validation cohort = 7.5	136	EP = 32, MB = 67, PA = 37	T1W, T2W, T1C, FLAIR, DWI (ADC)	Limited MR sequences, heterogeneity in image acquisition, standardisation of multiparametric MRI, use of anisotropic resampling, need for comparative studies to assess the added value of T1C based on ML
Fan et al.^14^	Differentiate between pineal region germinoma and pineoblastoma	6–19	134	Germinoma = 69, pineoblastoma = 65	T2W, T1C	Limited tumour region, lack of data from multiple centres, need for prospective patients.
Fetit et al.^15^	3D TA of heterogeneous MRI for BT classification	NA	121	EP = 18, MB = 42, PA = 61	Pre-contrast T1, T2W	Imbalanced dataset, need for cross-centre transferability of the model
Fetit et al.^16^	TA for BT classification	NA	134	EP = 18, MB = 45, PA = 71	T1W, T2W	Inclusion of synthesised samples generated by SMOTE in testing folds, heterogeneity in image acquisition, and lack of clear clinical meanings for identified imaging features as biomarkers
Fetit et al.^17^	3D TA of conventional MRI for BT classification	NA	48	EP = 7, MB = 21, PA = 20	T1W, T2W	Small sample size, heterogeneity in image acquisition, limited MR sequences, and lack of data from multiple centres
Grist et al.^18^	Classification of BTs by diffusion, perfusion, and ML	NA	49	EP = 10, MB = 17, PA = 22	T1W, T2W, T1CE, DWI, FLAIR, DSC	Small sample size, limited tumour region, heterogeneity in image acquisition.
Li et al.^19^	Differentiate between EP and PA	0–14	45	EP, PA	T1W, T2W	Limited feature selection and classifier evaluation, need for further validation
Novak et al,^20^	Classification of BTs by DWI and ML	1–16.3	124	EP = 26, MB = 55, PA = 36, others = 7	T1W, T2W, T1CE, DWI	Retrospective study design, limited tumour region, need for prospective validation, ADC unable to discriminate ATRTs from MB, manual segmentation susceptible to human error
Wang et al.^21^	Classify paediatric PFTs	4.99 ± 2.77	99	EP = 13, MB = 59, PA = 27	T1W, T2W, DWI (ADC)	Small sample size, retrospective study design, lack of molecular typing, variability in ROI selection, interpretability of radiomic features
Zhang et al.^22^	Differentiate ET, HGG, EP	0–18	231	EP = 54, ET = 50, HGG = 127	T2W, T1CE	Small sample size, heterogeneity in image acquisition, limited MR sequences, lack of data from multiple centres, limited pre-processing
Zhang et al.^23^	Differentiate ATRT from MB	1.0–114.6 months	144	ATRT = 48, MB = 96	T2W, T1CE	Small sample size, limited MR sequences, and lack of molecular data
Zhang et al.^24^	Classify PFTs	1.0–111.6 months	535	EP = 97, MB = 278, PA = 160	T1W, T2W	Small sample size, limited texture analysis
Zheng et al.^25^	Identification of CSF dissemination	<18	124	MB	T1W, CE-T1W	Small sample size, limited MR sequences, and lack of external validation
Zhou et al.^26^	Differentiate PFTs	0.25–18, mean = 8.6	288	EP = 70, MB = 111, PA = 107	T1C, T2W, DWI (ADC)	Limited MR sequences, lack of data from multiple centres, selection bias

**(b)**						
Study ref.	Image pre-processing	Segmentation technique	Feature extraction technique	Feature reduction and selection	Model	Best model performance [AUC (95% CI)]
Dong et al.^12^	N4 bias correction, isotropic resampling, signal intensity normalisation	3D semi-automatic	Histogram, shape, texture (GLCM, GLRLM, GLSZM)	UA, UAS, MLR	AdaBoost, k-NN, RF, SVM	0.910 (0.787–0.968)
Dong et al.^13^	N4ITK bias correction, anisotropic resampling, registration, signal intensity normalisation	Semi-automatic	Histogram, absolute gradient, shape, texture (GLCM, GLRLM)	FCBF	SVM	Primary: 0.941 (0.909–0.964). Validation: 0.977 (0.920–0.997)
Fan et al.^14^	NA	Semi-automatic	First-order, shape and size, texture (GLCM, GLDM, GLRLM, GLSZM), wavelet	UA, elastic net algorithm, RFE	SVM	Training: 0.950 (0.940–0.963). Validation: 0.940 (0.922–0.953)
Fetit et al.^15^	Normalisation	Semi-automatic	Histogram, absolute gradient, texture (GLCM, GLRLM)	Entropy-MDL discretisation	C-SVM	85%
Fetit et al.^16^	Normalisation	Semi-automatic	Histogram, absolute gradient, texture (GLCM, GLRLM)	Entropy-MDL discretisation, ReliefF	C-SVM	86%
Fetit et al.^17^	Slice selection, intensity normalisation	Semi-automatic	Histogram, absolute gradient, texture (GLCM, GLRLM)	Entropy-MDL discretisation	NB, k-NN, classification tree, SVM, ANN, LR	99%
Grist et al.^18^	Brain masking, registration, normalisation	Manual	Mean, standard deviation, skewness, kurtosis	PCA, UA	Single layer NN, AdaBoost, RF, SVM, k-NN	Precision = 86%
Li et al.^19^	NA	Manual	Histogram (GLH), texture (GLCM, GRLM, GGCM, Tamura, LJbG, Laws), IM	KWT	SVM	0.864
Novak et al.^20^	NA	Manual	Histogram metrics such as mean, median, variance, skew, kurtosis, quantiles	PCA	NB, RF	Accuracy = 86.3%
Wang et al.^1^	Image homogenisation, intensity normalisation, data balance	Manual	Histogram, texture (GLCM), wavelet	Feature ranking	RF	0.938
Zhang et al.^22^	Intensity normalisation, resampling	Manual	First-order, shape, texture (GLCM, GLRLM, GLSZM)	LASSO	SVM, LR, k-NN, RF, XGB, NN	0.98
Zhang et al.^23^	Normalisation, wavelet transform and edge detection	Manual	First-order, 2D/3D shape, grey-level matrices (GLCM, GLSZM, neighbouring grey-tone difference, dependence matrices)	LASSO	SVM, LR, k-NN, RF, XGB, NN	0.8582
Zhang et al.^24^	NA	Manual	IBSI-based features	LASSO	SVM, LR, k-NN, RF, XGB, NN	0.9243
Zheng et al.^25^	NA	Automatic	Histogram, volume and shape, Haralick, GLCM, GLRLM	mRMR, LASSO	Multivariable LR	0.89
Zhou et al.^26^	Normalisation	Manual	Texture	Normalisation and combination	SVM, TPOT	0.94

ADC: apparent diffusion coefficient; ATRT: atypical teratoid/rhabdoid tumour; BT: brain tumour; CE: contrast-enhanced; CSF: cerebrospinal fluid; DSC: dynamic susceptibility contrast; EP: ependymoma; ET: embryonal tumour; FLAIR: fluid-attenuated inversion recovery; HGG: high-grade glioma; MB: medulloblastoma; ML: machine learning; PA: pilocytic astrocytoma; PFT: posterior fossa tumour; T1C (or T1CE): T1-weighted contrast-enhanced; T1W: T1-weighted; T2W: T2-weighted; AdaBoost: adaptive boosting; ANN: artificial neural network; AUC (95% CI): area under the curve (95% confidence interval); C-SVM: C-support vector machine; Entropy-MDL Discretisation: entropy minimum description length discretisation; FCBF: fast correlation-based filter; GLCM: gray level co-occurrence matrix; GLDM: gray level dependence matrix; GGCM: gray level gradient co-occurrence matrix; GLH: gray level histogram; GLRLM: gray level run-length matrix; GLSZM: gray level size zone matrix; IBSI: image biomarker standardisation initiative; IM: image metrics; k-NN: k-nearest neighbour; LASSO: least absolute shrinkage and selection operator; LR: logistic regression; mRMR: minimum redundancy maximum relevance; MLR: multiple logistic regression; NB: Naïve Bayes; NN: neural network; NA: not available; PCA: principal component analysis; RFE: recursive feature elimination; RF: random forest; SVM: support vector machine; TPOT: tree-based pipeline optimisation tool; UA: univariate analysis; UAS: univariate analysis screening; XGB: xtreme gradient boosting.

**Table 3.** Radiomics approach for prognosis of paediatric brain tumours.

(a)						
Study ref.	Focus	Age range (years)	Sample size	Type of brain tumour	MR imaging sequence	Limitations
Liu et al.^27^	Prediction of PFS	1.0–18	253	MB	T1W, CE-T1W	Small sample size, retrospective study design, lack of molecular typing, and need for prospective validation
Tam et al.^28^	MRI-based radiomics prognosis of DIPG	1.6–19	177	DIPG	T1C, T2W	Small sample size, heterogeneity in image acquisition, lack of advanced imaging techniques, manual segmentation
Wagner et al.^29^	Prediction of PFS in paediatric DIPG	1–16.9	89	DIPG	T1C, T2W, FLAIR	Retrospective study design, heterogeneity in image acquisition, lack of external validation, imbalanced distribution of molecular subtypes, partial lack of biopsy data
Zheng et al.^30^	Risk stratification	Mean = 5.82	111	MB	T1CE with inversion recovery	Small sample size, lack of data from multiple centres, lack of molecular data

(b)						
Study ref.	Image pre-processing	Segmentation technique	Feature extraction technique	Feature reduction and selection	Model	Best model performance [AUC (95% CI)]
Liu et al.^27^	Image resampling, normalisation	Manual	First-order, GLCM, GLRM, GLSZM, neighbourhood grey-tone difference matrix, wavelet	Low-variance feature removal, correlation-based grouping, LASSO	X-tile, Kaplan Meier survival curve, log-rank test	C-indices of 0.711, 0.707, and 0.717 on the training and held-out test sets 1 and 2
Tam et al.^28^	NA	Manual	Size, shape, first-order, texture (original, wavelet, Laplacian of Gaussian)	LASSO	Multivariable Cox model	C-index 0.70 (0.64–0.77)
Wagner et al.^29^	Bias correction, normalisation	Semi-automatic	Histogram, shape, texture	Correlation	Conditional survival forest	C-index 0.87 (0.86–0.88)
Zheng et al.^30^	NA	Manual	First-order, volume and shape, texture (GLCM, GLRLM, GLSZM, GLDM)	Univariable Cox regression analysis, Pearson correlation, LASSO	Multivariable Cox regression analysis	C-index 0.823

CE-T1W: contrast-enhanced T1-weighted; DIPG: diffuse intrinsic pontine glioma; FLAIR: fluid-attenuated inversion recovery; MB: medulloblastoma; PFS: progression-free survival; T1C (or T1CE): T1-weighted contrast-enhanced; T1W: T1-weighted; T2W: T2-weighted; C-index: concordance index; GLCM: gray level co-occurrence matrix; GLDM: gray level dependence matrix; GLRM: gray level run-length matrix; GLRLM: grey-level run-length matrix; GLSZM: gray level size zone matrix; LASSO: least absolute shrinkage and selection operator; NA: not available.

Table 4 provides additional information on the extracted radiomic feature classes, their biological meaning, and clinical relevance.

**Table 4.** Feature classes, biological meaning, and their clinical relevance.

Feature class	Study ref.	Example	Explanation	Biological meaning	Clinical relevance
Texture	^12–17,19,20,22–24,28^	GLCM	Joint distribution of pixel greyscale values	Captures spatial relationships between pixel intensities providing information about tumour heterogeneity	Differentiates between various brain tumour types, subtypes, and grades. Also, aids in prognostic stratification
		GLRLM	Represents the length of uniform runs in various directions	Reflects the structural organisation of an image. It indicates texture regularity within a tumour	Based on texture characteristics, it assists in distinguishing between tumour types
		GLSZM	Information on size zones and their distribution	Shows texture consistency and non-periodicity. Information on the microstructure of the tumour	Improves understanding of heterogeneous tumours. Has the potential to guide treatment response and predictions
Shape	^12,14,19,22–25,28–30^	Volume	Measures the tumour region in cubic millimetres (mm^3^)	Shows size and extent of the tumour. Thus, indicates growth patterns and tumour burden	Tumour progression and response to treatment
		Surface area	Measures total area of the tumour boundary in square millimetres (mm²)	Information on tumour morphology and how it interacts with surrounding tissues	Surgical planning and tumour infiltration
Histogram	^12,15–21,25,26^	Mean intensity	Average intensity value within a tumour	Overall intensity distribution within the tumour	Quantifies intensity distribution, patterns, tumour phenotype characterisation
		Skewness	Measures the asymmetry of the intensity distribution	Asymmetry within a tumour	Characterises distribution patterns may indicate necrosis or tumour complexity
		Variance	Measures the spread of voxel intensities	Variations in voxel intensities	Insights into tumour heterogeneity, it complements texture-based features
Wavelet	^14,21,27^	Wavelet decomposition	Captures multiscale texture patterns at different levels of image resolution	Identifies tumour heterogeneity across scales	Used in classification and prognosis by detecting both fine and coarse texture patterns
Additional features	^13,15,17^	Absolute gradient	Rate at which intensity changes within the image	Provides detailed information on sharp transitions between different tissue regions	Helps in differentiating tumour shapes regardless of their size and orientation

GLCM: grey-level co-occurrence matrix; GLRLM: grey-level run-length matrix; GLSZM: grey-level size zone matrix.

### Evaluation metrics

In this systematic review, the best model performance from each selected study was recorded, expressed as the area under the receiver operating characteristic curve (AUC) (unless otherwise specified) which evaluates a model's diagnostic performance integrating both sensitivity and specificity across varying classification thresholds. A higher AUC represents superior discriminative ability and reporting a 95% confidence interval (CI) around the AUC also helps with comparisons between different radiomics models or study findings. Other metrics were also reported in certain studies such as accuracy (proportion of all correctly classified cases), sensitivity/recall (ability to correctly identify positive instances), specificity (capacity to accurately rule out negative cases), precision (proportion of predicted positives that are true positives), and F1-score (harmonic mean of precision and recall).

### Radiomics in paediatric neuro-oncology

The distribution of studies focused on various types of paediatric brain tumours such as medulloblastoma (MB) (11), ependymoma (EP) (5), diffuse intrinsic pontine glioma (DIPG) (2), pineal region germinoma (1), pineoblastoma (1), along with six studies covering other brain tumour types, including astrocytomas, gliomas, and unspecified tumours. Sample sizes varied across studies and ages ranged from infants (<1 month old) to 18 years old.

#### Diagnosis

Fifteen studies were diagnosis focused. Table 2(a) and (b) lists key characteristics of each study, including age, sample size, tumour type, imaging modality, image pre-processing techniques, segmentation approach, feature extraction and selection methods, and best model performance.

A thorough analysis of the selected studies revealed the use of a diverse range of radiomic approaches to extract quantitative features from images, and ML techniques that use algorithms to analyse these features and develop predictive models for the diagnosis of paediatric brain tumours. Dong et al.^12^ differentiated EP and MB, with an AUC of 0.910 using identified imaging biomarkers, while Dong et al.^13^ classified Paediatric Posterior Fossa Tumours (PPFTs), with an AUC of 0.977 by extracting features from multiparametric MRI. Replacing contrast-enhanced T1W images with apparent diffusion coefficient (ADC) maps improved performance, highlighting the importance of using additional MRI sequences. Similarly, ML's efficacy in PPFT differentiation was also studied by^19^ targeting pre-operative differentiation between EP and PA. Multicentre use of ADC for classifying PFTs achieved high accuracy across diverse hospital settings and scanners.^20^ Wang et al.^21^ also classified PPFTs, achieving 93.8% accuracy using a random forest (RF) classifier. It is important to note that, the best features for classification came from the ADC sequence. This highlights the value of ADC sequences in extracting quantitative information in differentiating between different types of childhood brain tumours. Zhang et al.^22^ also used ML to differentiate embryonal tumours (ETs), High-Grade Gliomas (HGGs), and EPs in paediatric supratentorial tumours. Zhang et al.^23^ used radiomic phenotypes derived from T2 W and gadolinium-enhanced T1W MRI images to distinguish atypical teratoid/rhabdoid tumours from MBs. Zhang et al.^24^ proposed a sequential ML classifier to improve the diagnosis of PPFTs across multinational cohorts. Zhou et al.^26^ also focused on differentiated different types of PPFTs using ML approaches applied to routine MR imaging. They compared manual expert optimisation with automatic ML using the Tree-Based Pipeline Optimisation Tool. The automatic ML model achieved significantly higher accuracy in classification.

To differentiate between pineal region germinoma and pinealoblastoma, a clinic–radiomic model was used.^14^ Using both radiomic and clinical features, the model achieved high discrimination with an AUC of 0.950 in the training set and 0.940 in the validation set. This non-invasive approach demonstrated high sensitivity and performed well in differentiating pineal region tumours.

Studies^15–17^ focused on improving the diagnostic classification of childhood brain tumours using texture analysis (TA). While Fetit et al.^15^ conducted a multicentre study and achieved good model performance across different centres and scanners, Fetit et al.^16^ evaluated the efficacy of three-dimensional (3D) TA compared to 2D TA, showing improved classification performance with 3D textural features. Fetit et al.^17^ assessed the efficacy of both 3D TA and 2D TA, re-confirming classifiers trained with 3D textural features show improved classification performance compared to those trained with conventional 2D features alone.

The integration of multicentre diffusion and perfusion imaging with ML resulted in a classifier with >80% predictive precision.^18^ Finally, in Zheng et al.,^25^ multivariable logistic regression was used to combine clinical and radiomic features for prediction of pre-operative cerebrospinal fluid (CSF) dissemination in children with MB. Combining one clinical and nine radiomic features demonstrated higher predictive performance with an AUC of 0.89.

#### Prognosis

Four studies were prognosis-focused. Table 3(a) and (b) summarises key characteristics and results for each study.

In Liu et al.,^27^ a radiomics signature and nomogram were developed to predict progression-free survival (PFS) in MB patients. It can be used to stratify patients for postoperative radiotherapy. Tam et al.^28^ focused on DIPGs, using MRI-based radiomics to create a prognostic model for overall survival (OS), which outperformed other models that were based only on clinical variables. In contrast, Wagner et al.^29^ evaluated the prognostic value of radiomics in DIPG subsets, demonstrating the potential of MRI-based radiomics in progression-free survival. Finally, in predicting overall survival in MB patients, Zheng et al.^30^ highlighted that an integrative model combining radiomics, clinical, and conventional MRI features, outperformed other models. All these studies showed emphasis on the prognostic value of radiomics in paediatric brain tumours.

#### Feature classes, biological meaning, and clinical relevance

Based on the studies, texture-based features^12–17,19,20,22–24,28^ were seen as a preferred choice for brain tumour classification and characterisation, because of their higher discriminative power and ability to capture fine-grained spatial patterns, and variations within the tumour region. These patterns are believed to be biologically linked to cellular variability, angiogenesis, and necrosis, which are all important for tumour progression and understanding response to treatment.^33^ Thus, they offer a more comprehensive and detailed characterisation of tumour heterogeneity and microstructure, compared to histogram-based features.

In contrast, histogram-based features^12,15–21,25,26^ primarily describe the overall distribution of voxel intensities without considering spatial relationships or textural patterns. Features, such as skewness and variance,^12,15,21,25,26^ describe the variability of intensities within a tumour, which may reflect the areas of necrosis or differing tissue densities, both of which have biological and prognostic relevance.^34^

Shape-based features^12,14,19,22–25,28–30^ provide information about tumour morphology, invasion, and geometry. These correlate with the aggressiveness of a tumour and thus have an impact on surgical planning and treatment decisions. However, they may not always capture the subtle differences in tissue texture and composition captured by texture features.^34^

#### Challenges

Various challenges associated with MRI-based radiomics in the diagnosis and prognosis of children's brain tumours were identified across studies, as listed in Tables 2(a) and 3(a). The most frequently reported limitation was a small sample size, highlighted in 14 studies. Additionally, the use of limited MR sequences, heterogeneity in image acquisition, and retrospective study design were prevalent, mentioned in 10 studies. Imbalanced datasets, lack of data from multiple centres, and the need for prospective validation were also commonly cited limitations, identified in seven studies each.

## Discussion

It has been seen in this review that in the context of diagnosis, radiomics and ML techniques have been widely used to improve the classification of various types of paediatric brain tumours. Studies^12,13,21^ showed how effective ML algorithms are in accurately differentiating EP, MB, and other PPFTs. The integration of advanced MRI sequences, particularly ADC maps, was seen as one of the most important factors in enhancing diagnostic accuracy.^13,20^ The use of ML models across multiple centres highlights their robustness and potential for real-world clinical application.^19,26^ Also, the integration of radiomic and clinical features outperformed conventional models in brain tumour characterisation.^14^

In the prognosis-focused studies, the prognostic value of radiomics in predicting PFS and OS, in paediatric brain tumour patients, was seen. The development of radiomics signatures, which quantify imaging features to predict tumour behaviour, and nomograms, which visually represent statistical models for estimating patient outcomes, provides valuable tools for stratifying patients and guiding treatment decisions.^27,28,30^ Integrating radiomic (e.g. texture and shape), clinical (e.g. patient demographics and laboratory results), and conventional MRI features (e.g. tumour morphology and anatomical information) in prognostic models, showed improved performance compared to models based only on clinical variables,^30^ emphasising the importance of a multimodal approach to prognostic assessment.

Radiomic features quantitatively describe tumour characteristics such as shape, texture, and intensity.^8,33^ In this review, the biological meaning of these features is also mentioned to understand the underlying biological mechanisms that these features reflect, which is essential for easier clinical translation of radiomics (Table 4). These image characteristics are also used to see if they can be connected to a disease status (helpful in the prediction of an outcome).

However, the data-driven nature of radiomics and the heavy use of ML techniques do not provide insights into the biological mechanisms of these observed relations. This often leads to slower translation into clinical practice as the biological meaning and interpretation of these features are often missing.^8,33^ This is an attempt to reconnect radiomic findings to biological meaning.

Features such as texture, shape, histogram, and wavelet reveal additional information about tumour biology and can be used to accurately classify tumour types, subtypes, and grades. Texture features such as GLCM, GLRLM, and GLSZM reveal patterns and spatial relationships within the image, showing consistency or heterogeneity of the tumour.

However, despite these advancements, challenges still exist. To mitigate the identified challenges effectively, innovative solutions are essential. For example, collaborative efforts among multiple centres can facilitate data pooling, overcoming limitations such as small cohorts and enabling validation across diverse patient populations.^15,20,22,26^ Integrating advanced imaging techniques and molecular data into radiomic analysis can improve the accuracy and robustness of models^13,20,23,27–30^ and make them more generalisable. Additionally, standardising imaging protocols and using fully automatic segmentation techniques can reduce image acquisition variability and enhance the reproducibility of the radiomics approach used.

With the rise in the integration of artificial intelligence, multi-omics data, and novel imaging protocols, new challenges have emerged. These include interpretability of increasingly complex models, data governance and regularity oversight, data storage issues, and computational infrastructure for real-time analyses, among others.^35^ Addressing these issues will require cross-disciplinary collaborations, the development of secure data-sharing and storage platforms, and refining ethical frameworks.

The majority of radiomics-related studies in paediatric neuro-oncology including the papers included in this review primarily discuss algorithmic approaches (such as segmentation, feature extraction, and ML) and workflow optimisation (such as standardising pipelines). None explicitly discuss hardware implementation strategies. Yet, hardware considerations are equally important when improving computational workflows, especially given the large volume of high-resolution MRI data and the complexity of radiomic pipelines. In general, using graphics processing units (GPUs) and high-performance computing (HPC) environments, including clusters and cloud-based solutions, when performing radiomics computations can accelerate data processing, and significantly reduce processing times for feature extraction and model training.^36^ However, specialised hardware comes at a cost, especially for smaller research groups and healthcare institutions with limited resources. Also, different hardware setups could lead to variations in performance and reproducibility. It is therefore necessary to report hardware specifications and computational runtimes to facilitate replication and comparison across studies.

While previous reviews^37,38^ have significantly advanced our understanding of radiomics and artificial intelligence in paediatric neuro-oncology, our review extends this foundation as it includes not only a detailed analysis of the entire radiomics pipeline but also considers the biological meaning of radiomic features and discusses emerging challenges such as hardware implementation. This offers a comprehensive and clinically relevant perspective to guide future research in the field.

## Conclusion

This systematic review highlighted key techniques used in all the phases of a radiomics pipeline, including image pre-processing, segmentation, feature extraction, feature selection and reduction, and model building. It also highlighted the biological meaning and clinical relevance of the extracted radiomic features. The identification and integration of the most informative radiomic features are important, as they hold the potential to significantly improve diagnostic and prognostic accuracy. Research should focus on validating and standardising these features to facilitate easier translation into clinical practice.

Despite advancements in radiomics, challenges such as limited datasets and inconsistent image acquisition protocols still exist. Addressing these challenges will further enhance radiomic analysis. Data-sharing among institutions and the use of transfer learning to adapt models trained on larger datasets to smaller, more specialised datasets, such as paediatric brain tumours, are recommended. Ultimately, refining radiomic analysis, using more generalisable models, and integrating them with clinical data will be essential in advancing the field of paediatric neuro-oncology.

## Supplemental Material

sj-docx-1-dhj-10.1177_20552076251336285 - Supplemental material for Magnetic resonance imaging (MRI) radiomics in paediatric neuro-oncology: A systematic review of clinical applications, feature interpretation, and biological insights in the characterisation and management of childhood brain tumours Supplemental material, sj-docx-1-dhj-10.1177_20552076251336285 for Magnetic resonance imaging (MRI) radiomics in paediatric neuro-oncology: A systematic review of clinical applications, feature interpretation, and biological insights in the characterisation and management of childhood brain tumours by Teesta Mukherjee, Omid Pournik and Theodoros N Arvanitis in DIGITAL HEALTH

## Abbreviations

3D: three-dimensional
AdaBoost: adaptive boosting
ADC: apparent diffusion coefficient
ANN: artificial neural network
ATRT: atypical teratoid rhabdoid tumours
AUC: area under the curve
BT: brain tumour
C-index: concordance index
CI: confidence interval
CSF: cerebral spinal fluid
CT: computed tomography
DIPG: diffuse intrinsic pontine glioma
EP: ependymoma
ET: embryonal tumour
FCBF: fast correlation-based feature
FLAIR: fluid-attenuated inversion recovery
GGCM: grey-level grouping co-occurrence matrix
GLCM: grey-level co-occurrence matrix
GLDM: grey-level dependence matrix
GLH: grey-level histogram
GLRLM: grey-level run-length matrix
GLSZM: grey-level size zone matrix
HGG: high-grade glioma
IBSI: image biomarker standardisation initiative
IM: invariant moment
k-NN: k-nearest neighbours algorithm
KWT: Kruskal–Wallis test
LASSO: least absolute shrinkage and selection operator
LJbG: local jet-based grey-level statistic
LR: logistic regression
MB: medulloblastoma
MDL: minimum descriptive length
ML: machine learning
MLR: multivariable logistic regression
MR: magnetic resonance
MRI: magnetic resonance imaging
mRMR: minimum redundancy maximum relevance
MR: magnetic resonance
N4: nonparametric nonuniform intensity normalisation
NB: Naïve Bayes
PCA: principal component analysis
PFS: progression-free survival
PFT: posterior fossa tumour
PPFT: paediatric posterior fossa tumour
PRISMA: preferred reporting items for systematic reviews and meta-analyses
PROSPERO: prospective register of systematic reviews
RFE: recursive feature elimination
SVM: support vector machine
TA: texture analysis
T1C: T1-weighted post-contrast
T1W: T1-weighted
T2W: T2-weighted
TPOT: tree-based pipeline optimisation tool
UA: univariate analysis
UAS: univariate analysis screening
XGB: extreme gradient machine
